# Application of Nanogenerators in Lumbar Motion Monitoring: Fundamentals, Current Status, and Perspectives

**DOI:** 10.3390/diagnostics15202657

**Published:** 2025-10-21

**Authors:** Yudong Zhao, Hongbin He, Junhao Tong, Tianchang Wang, Shini Wang, Zhuoran Sun, Weishi Li, Siyu Zhou

**Affiliations:** 1 Orthopaedic Department, Peking University Third Hospital, No. 49 North Garden Road, Haidian District, Beijing 100191, China; zydddg99@163.com (Y.Z.); puh3_szr@163.com (Z.S.); 2 Orthopedic Department, Peking University Third Hospital, Peking University Health Science Center, No. 38 Xueyuan Road, Haidian District, Beijing 100191, China; 2310301136@stu.pku.edu.cn (H.H.); 2410122509@stu.pku.edu.cn (J.T.); 2410301110@stu.pku.edu.cn (T.W.); 2310301112@stu.pku.edu.cn (S.W.); 3 Beijing Key Laboratory of Spinal Disease Research, No. 49 North Garden Road, Haidian District, Beijing 100191, China; 4 Engineering Research Center of Bone and Joint Precision Medicine, Ministry of Education, No. 49 North Garden Road, Haidian District, Beijing 100191, China

**Keywords:** nanogenerators, lumbar, motion monitoring, low back pain

## Abstract

Nanogenerators (NGs), especially triboelectric nanogenerators (TENGs), represent an emerging technology with great potential for self-powered lumbar motion monitoring. Conventional wearable systems for assessing spinal kinematics are often limited by their reliance on external power supplies, hindering long-term and real-time clinical applications. NGs can convert biomechanical energy from lumbar motion into electrical energy, providing both sensing and power-generation capabilities in a single platform. This review summarizes the fundamental working mechanisms, device architectures, and current progress of NG-based motion monitoring technologies, with a particular focus on their applications in lumbar spine research and clinical rehabilitation. By enabling high-sensitivity, continuous, and battery-free monitoring, NG-based systems may enhance the diagnosis and management of low back pain (LBP) and postoperative recovery assessment. Furthermore, the integration of NGs with wearable electronics, the Internet of Things (IoT), and artificial intelligence (AI) holds promise for developing intelligent, self-sustaining monitoring platforms that bridge biomedical engineering and spine medicine.

## 1. Introduction

Since the advent of thermal power generation, the primary method for humans to obtain electricity has been the combustion of fossil fuels. However, the search for new, efficient, and abundant energy conversion systems was always paramount [1]. Following the introduction of the first piezoelectric nanogenerator (PENG) in 2006 [2], numerous studies have subsequently updated the understanding of this novel generator in terms of materials and mechanisms [3,4]. Nanogenerators (NGs) are devices capable of converting mechanical energy into electrical energy. Their working principles vary depending on the type, encompassing the piezoelectric effect, electrostatic effect, thermoelectric effect, among others [5,6,7,8,9].

**Figure 1:**
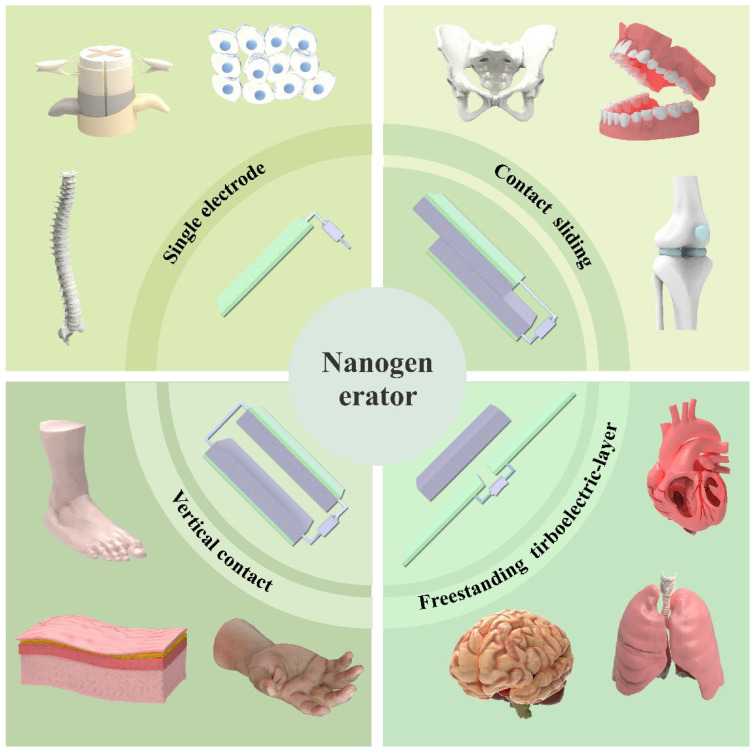
The main types of nanogenerators.

Triboelectric nanogenerators (TENGs), operating through the coupling of contact electrification and electrostatic induction, excel in various scenarios. Their fundamental principle involves the periodic contact and separation of two materials with opposing triboelectric polarities, which drives the alternating flow of induced electrons between electrodes [10,11], thereby achieving mechanical-to-electrical energy conversion [1]. Compared to other nanogenerators, TENGs offer advantages such as lower cost, higher efficiency, and greater power density [12], making them a focal point in nanogenerator research. To date, four primary types of TENGs have been established (Figure 1): vertical contact-separation mode, lateral sliding mode, single-electrode mode, and free-standing triboelectric-layer mode [10,13,14,15,16,17,18,19], each possessing distinct characteristics and advantages, with vertical contact-separation mode being the most extensively studied [5].

Throughout their development, nanogenerators have progressively demonstrated significant application potential across diverse fields, including environmental science and medicine [20,21,22], achieving notable progress. Initially hindered by low energy conversion efficiency and rudimentary fundamental principles, they saw limited widespread practical application. However, the emergence of flexible TENGs has facilitated the design of wearable devices, further advancing the development of human motion monitoring technology [23].

In this paper, we reviewed the importance and current application of lumbar motion monitoring, also giving a perspective on how TENG could be further applied in lumbar motion monitoring. This review aims to bridge the gap between advanced nanogenerator technologies and their clinical translation in lumbar motion monitoring.

## 2. Significance of Lumbar Motion Monitoring and Basic Components of Monitoring Devices

Lumbar diseases exhibit high prevalence in the population. For example, low back pain (LBP) affects approximately 80–85% of individuals at least once in their lifetime, prompting them to seek medical attention [24]. Disease-related LBP often severely impacts patients’ quality of life. Although the origins of LBP are diverse: abnormalities in muscles, intervertebral discs, vertebral bodies and spinal alignment, etc., its relationship with lumbar mobility is intrinsically linked: LBP restricts lumbar movement, while declines in lumbar mobility, alongside LBP, can reflect the underlying etiology [25,26]. Conservative treatments, such as osteopathic manipulative treatment (OMT), are commonly employed for managing low back pain (LBP). A recent systematic review by Dwornik et al. (2024) [27] indicated that OMT can effectively reduce pain and improve functional status in patients with non-specific LBP, particularly in musculoskeletal disorders. However, the efficacy of such interventions relies on accurate and dynamic assessment of lumbar mobility, which is often limited by conventional static imaging methods. This underscores the need for advanced monitoring technologies that can provide continuous, real-time data to support personalized rehabilitation strategies. Also, current diagnostic approaches for lumbar disorders are trending from static towards dynamic assessments [28]. Dynamic lumbar mobility evaluation often involves flexion-extension X-rays taken in a hospital setting but it is limited by the patient’s condition at the time of imaging and only captures a single time point [29]. In contrast, wearable lumbar monitoring devices enable continuous monitoring of patient status to give detailed analysis of patient’s lumbar movement.

### 2.1. Basics of Lumbar Motion Monitoring Devices

Lumbar motion monitoring devices typically consist of sensors, a data processing unit, and a feedback module. Sensors detect motion information such as acceleration and angular changes. For example, the VitaMove activity monitor, a high-end accelerometer-based device, automatically detects a wide range of body postures and movements, providing detailed assessments including the proportion of time spent in different postures and activities, sit-to-stand transitions, overall activity levels, walking speed, and the distribution of physical activity and sedentary behavior [30,31,32]. The data processing unit analyzes and processes the collected sensor data to extract valuable information. The feedback module then presents the processed information intuitively to the user, such as visualizing lumbar motion parameters via an interface, helping users understand their lumbar status. Some devices also incorporate real-time feedback, issuing alerts upon detecting abnormal movements. For instance, an ultrasound-based automatic patient motion monitoring device can detect movement during treatment using ultrasound transmitters and receivers, activate a circuit to sound an alarm, and simultaneously interrupt the therapy machine’s circuit to halt radiation, thereby ensuring treatment accuracy and preventing unnecessary radiation exposure to healthy tissue [33].

Current methods are diverse, including devices based on inertial measurement units (IMUs), pressure sensors, and visual monitoring systems [34,35]. IMU-based devices acquire lumbar motion information by measuring acceleration and angular velocity. For example, the DorsaVi wireless motion sensor system demonstrated acceptable error margins compared to the Vicon motion analysis system when measuring lumbar flexion/extension during symmetric and asymmetric lifting tasks, indicating its reliability for lumbar motion monitoring [36]. Pressure sensors monitor lumbar motion by detecting pressure changes, such as wearable pressure-sensing belts that analyze movement state alterations based on variations in lumbar force exertion. However, discrepancies in measured physical activity parameters exist depending on sensor placement (e.g., wrist vs. waist placement of ActiGraph accelerometers), highlighting the need to consider sensor location for measurement accuracy [37]. Visual monitoring systems use cameras to record lumbar motion, but may raise privacy concerns and are susceptible to environmental influences. Additionally, monitoring techniques based on ultrasound, optics, etc., exist [38], each with its own advantages.

### 2.2. Lumbar Motion Monitoring and Lumbar Spine Disorders

**Figure 2:**
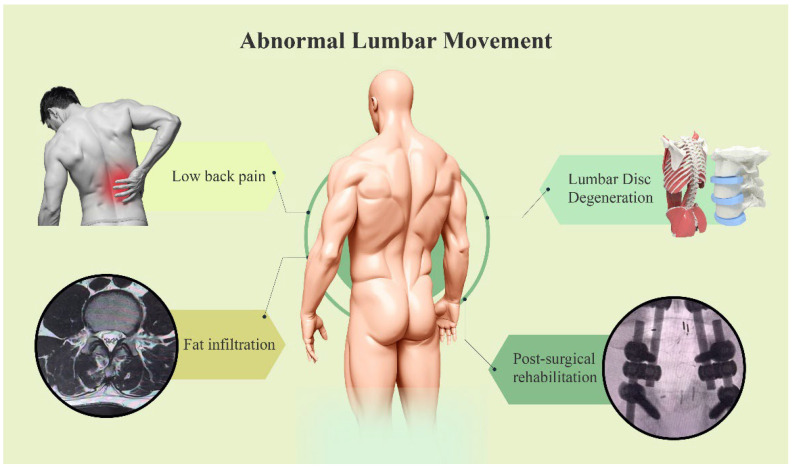
The relationship between abnormal lumbar movement patterns and lumbar diseases.

Abnormal lumbar motion demonstrated a close relationship with lumbar disorders (Figure 2). A study comparing lumbar motion consistency between LBP patients and pain-free individuals found significant differences in movement patterns under various test conditions in the LBP group [39]. Therefore, monitoring lumbar motion is essential for understanding the pathophysiological mechanisms of LBP, developing personalized treatment plans, and evaluating treatment efficacy. In specific clinical applications, physicians and therapists can utilize lumbar monitoring devices to understand a patient’s lumbar motion status, enabling the formulation of more personalized rehabilitation plans [40,41]. For high-risk populations, such as those engaged in prolonged sedentary work or physical labor, wearable lumbar motion monitors can provide real-time reminders to maintain correct posture and movement patterns, preventing lumbar injuries. Concurrently, long-term monitoring and analysis of daily lumbar motion data can help identify potential lumbar problems early, allowing for timely intervention to reduce LBP risk. For example, a study comparing the ViMove sensor system with the Vicon motion capture system found high accuracy in ViMove for measuring lumbar inclination motion, with small root mean square errors and good agreement within 95% confidence intervals [42].

Lumbar motion monitoring is also valuable for studying paraspinal muscle degeneration. Degeneration, characterized by reduced cross-sectional area, muscle fiber atrophy, and fatty infiltration, often leads to weakened muscle strength, resulting in lumbar stiffness and tightness, restricted range of motion, and decreased spinal stability. Research indicates that repetitive movements or sustained postures in specific directions may contribute to pathological changes in relevant tissues [43]. Analyzing lumbar motion allows for both accurately measuring the impact of paraspinal muscle degeneration and investigating which specific movement patterns strongly correlate with muscle degeneration, proving crucial for studying paraspinal muscle degeneration [44]. For instance, combining surface electromyography (sEMG) with lumbar kinematic measurements can elucidate the relationship between muscle activity and spinal movement, providing insights into the pathological mechanisms of lumbar disorders. Monitoring has revealed that abnormal activation patterns of lumbar muscles in some chronic LBP patients may lead to decreased spinal stability, exacerbating their condition [45]. For patients undergoing lumbar spine surgery, pre- and post-operative lumbar motion monitoring can assess surgical outcomes and rehabilitation progress. For example, a study on lumbar surgery patients, through long-term monitoring of pre- and post-operative lumbar motion, found that kinesiophobia (fear of movement) was associated with the level of LBP-related disability up to two years post-surgery, suggesting that addressing kinesiophobia during rehabilitation can improve health outcomes [46].

## 3. Integration of Nanogenerator Technology and Lumbar Motion Monitoring

Nanogenerators offer unique advantages for lumbar motion monitoring. Their self-powering capability eliminates the need for frequent battery replacements in monitoring devices, enhancing user convenience and long-term stability. Some nanogenerator-based wearable devices can continuously harvest energy from human motion to power monitoring functions, enabling prolonged, uninterrupted monitoring of lumbar motion [47]. For instance, an NG-based electronic skin can collect mechanical energy from body movement, convert it into electricity, and power sensors and data processing modules for lumbar motion monitoring [48]. Moreover, the electricity harvested by NGs can not only power the monitoring device itself but also supply other smart systems related to lumbar motion. By combining Internet of Things (IoT) and Artificial Intelligence (AI), it can establish a more useful self-sustaining energy loop [49].

Recent progress in material science has significantly advanced the performance of nanogenerators, particularly in flexibility, stability, and electrical output. A notable innovation is the development of semi-interpenetrating polymer network (semi-IPN) hydrogels, which combine high strength and conductivity, addressing the traditional trade-off between mechanical robustness and electrical performance in soft materials [50]. Similarly, Wang et al. demonstrated the use of fully polymeric conductive hydrogels, which exhibit exceptional stretchability (~1000%), low hysteresis, and reliable conductivity, making them highly suitable for use as electrodes in TENGs [51]. These advances are crucial in developing wearable, skin-conformal, low-noise devices for continuous lumbar motion monitoring, as they ensure high output and durability under dynamic, real-world conditions. This work underscores the versatility of cellulose in wearable TENGs, which could also be adapted for lumbar motion sensors, providing sustainable and efficient energy harvesting from human movements.

In addition to single-point or small-scale sensors, large-area and multi-node sensing platforms are emerging as essential for capturing complex spinal movements. For instance, Shao et al. recently demonstrated an omnidirectionally stretchable, self-powered electronic skin (UTE-skin) capable of large-area, high-fidelity tactile sensing [52]. This technological advance is pivotal for lumbar motion monitoring, where dynamic, multi-segmental, and multidirectional movements must be accurately assessed. By employing a distributed, multi-node TENG-based electronic skin, it is possible to monitor the spine’s full range of motion in real-time, providing continuous, comprehensive data crucial for rehabilitation and health diagnostics. Furthermore, Park et al. emphasized the importance of integrating soft sensors and actuators into wearable human–machine interfaces (HMIs), which provide tactile feedback through stretchable materials [53]. This principle is directly applicable to lumbar motion monitoring, where such soft actuators could offer real-time feedback about the user’s posture or motion, enhancing both diagnostics and user experience. These advances in sensor integration ensure that TENGs not only harvest energy but also provide essential feedback, facilitating the development of self-powered, interactive lumbar motion systems.

**Figure 3:**
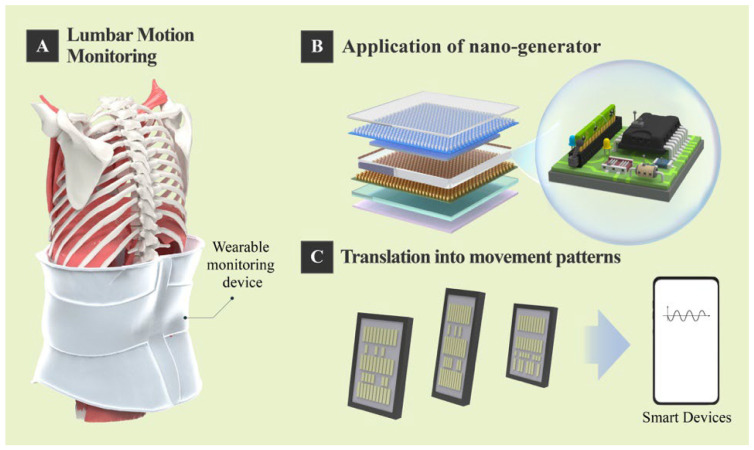
Application of lumbar motion monitoring device based on nanogenerator.

The integration of nanogenerators with the Internet of Things (IoT) and Artificial Intelligence (AI) is paving the way for a transformative paradigm in lumbar motion monitoring—shifting from episodic assessment to continuous, intelligent, and personalized healthcare (Figure 3) [54]. Wearable NG sensors can serve as self-powered nodes within an IoT network. For instance, Zhang et al. developed a textile-based triboelectric sensory system that wirelessly transmits real-time gait and waist motion data to a cloud platform via Bluetooth modules [55]. This enables continuous remote monitoring by clinicians and long-term data accumulation for longitudinal analysis. Such an IoT architecture facilitates timely interventions and tele-rehabilitation, which is particularly valuable for patients with chronic low back pain (CLBP) or those in post-operative recovery. On the other hand, the high-dimensional data generated by NG sensors are ideal for AI analysis. Machine learning (ML) and deep learning (DL) models can extract meaningful patterns from kinematic data to support clinical decision-making. Zhang et al. achieved 98.4% accuracy in identifying individuals based on their unique gait patterns using an artificial neural network (ANN) [55], allowing for automatic selection of personalized rehabilitation plans. AI algorithms can learn from continuous motion data to predict exacerbation risks, classify movement impairment subtypes (e.g., flexion- or extension-based LBP), and provide real-time biofeedback to correct harmful movement patterns [55,56,57]. Furthermore, multi-view clustering methods based on machine learning have shown robustness in handling motion data from different perspectives, even in the presence of noise, which enhances the reliability of community-based sports and rehabilitation monitoring [58].

## 4. Future Perspectives

As triboelectric nanogenerators (TENGs) continue to advance, future lumbar motion monitoring systems will benefit from the integration of next-generation materials and smart technologies.

Recent developments in semi-interpenetrating polymer network (semi-IPN) hydrogels and fully polymeric conductive hydrogels have greatly improved TENG’s energy conversion efficiency and mechanical performance, making them ideal candidates for wearable applications. Han et al. (2024) demonstrated how semi-IPN hydrogels can combine high conductivity with mechanical robustness, which is crucial for the durability and efficiency of self-powered wearable devices in real-world conditions [50]. Similarly, Wang et al. (2024) introduced polymeric hydrogels with exceptional stretchability and low hysteresis, which could revolutionize the design of flexible, skin-conformal lumbar motion sensors [59]. These innovations are setting the stage for long-term, non-invasive lumbar health monitoring with high output performance, which is essential for both clinical and personal applications.

Simultaneously, the development of hybrid nanogenerator systems, which combine TENGs with other materials or power sources, could provide enhanced power output and increased reliability for long-term use. Fan et al. (2016) explored the potential of combining piezoelectric and triboelectric materials to create hybrid energy harvesters, which could significantly improve the performance of wearable sensors in challenging environments [21]. A composite triboelectric nanogenerator (CTENG) fabricated by adding barium titanate (BTO) nanopowder and graphene quantum dots (GQDs) to a polydimethylsiloxane (PDMS) polymer matrix showed significantly enhanced output performance at a GQD concentration of 30 wt%. The open-circuit voltage (VOC), short-circuit current (ISC), and power density reached approximately 310.0 V, 23.0 μA, and 1.6 W/m^2^, respectively [60]. Another study demonstrated that the dielectric constant of ZnS/PDMS increased with rising PANI concentration, gradually enhancing the electrical output of a hybrid piezoelectric-triboelectric nanogenerator (HPTNG). The HPTNG based on PANI2.5-ZnS/PDMS exhibited high electrical outputs of 180 V and 280 μW/cm [61]. Wearable nanogenerators combining hydrogel materials have already demonstrated capabilities such as powering 52 light-emitting diodes [54]. Whether involving single or composite materials, the fundamental goal is to enhance NG output performance. These material innovations improve energy conversion efficiency while making NGs more portable, precise, efficient, and better suited for clinical applications in human motion monitoring.

Some new studies show that in piezoelectric polymer textiles, post-stretching and annealing treatments of nylon-11,11 textiles significantly enhance their piezoelectric performance. After treatment, the remnant polarization (Pr) of nylon-11,11 textiles increased by 4.7 times compared to untreated textiles. The resulting piezoelectric nanogenerator (PENG) achieved output voltage, current, and power density of 21.5 V, 800 nA, and 1.88 mW·m^−2^ (at 80 MΩ), respectively. Its mechanical voltage and current sensitivities reached as high as 266 mV/kPa and 13.99 nA/kPa under pressures exceeding 8 kPa, indicating potential for further PENG performance improvement [62].

The market demand for lumbar motion monitoring devices is growing. Rising health awareness and increasing concern about lumbar health, particularly among populations engaged in prolonged sedentary work or susceptible to lumbar injuries (e.g., office workers, athletes), drive a strong demand for devices capable of real-time monitoring and prevention. These devices also have a broad market in medical rehabilitation, providing vital evidence for treatment planning and outcome assessment. However, the market faces challenges. Firstly, while numerous devices exist, their quality varies, and the accuracy and reliability of monitoring data need improvement. Significant discrepancies in measurements between different brands and types create confusion for users [63]. Secondly, device comfort and portability require attention; bulky or uncomfortable devices can deter usage. With the further development of NG technology and its application in lumbar motion monitoring, smaller, more convenient, and more precise monitoring devices are expected to find wider application prospects.

## 5. Conclusions

This review outlines the significant potential of nanogenerators (NGs), particularly triboelectric NGs (TENGs), as a core technology for next-generation lumbar motion monitoring. Their self-powering capability, flexibility, and high sensitivity address key limitations of current methods, enabling continuous, wearable assessment of lumbar biomechanics. The integration of NG-based sensors with IoT and AI platforms promises a future of intelligent, real-time health monitoring and personalized feedback, which is crucial for managing conditions like low back pain. However, clinical translation requires further progress in material stability, device integration, and validation through large-scale studies. Ultimately, NG technology is poised to bridge the gap between biomechanical sensing and conservative clinical management, offering a powerful tool for both diagnosis and rehabilitation of lumbar disorders.

A key strength of this review is its interdisciplinary synthesis, connecting advances in materials science and NG engineering with concrete clinical applications in lumbar care, providing a clear vision for future development. However, it focused less on technological advances in TENG. Also, as a narrative review, it may be subject to selection bias due to the non-systematic literature search methodology. Furthermore, the conclusions are necessarily forward-looking, as many of the discussed applications are still in the laboratory stage, with a clear gap between technological proof-of-concept and validated clinical use.

## Data Availability

The data that support the findings of this study are available from the corresponding authors and Y.D. Zhao upon reasonable request.
